# Endoscopic-assisted descending aortic replacement through a small intercostal incision: a case report

**DOI:** 10.1186/s13019-022-02059-3

**Published:** 2022-12-12

**Authors:** Takeshi Wada, Hidenori Sako, Kenya Kizu, Takahiro Tashima, Tetsushi Takayama, Shinji Miyamoto

**Affiliations:** 1 Department of Cardiovascular Surgery, Oita Oka Hospital, 3-7-11, Nishitsurusaki, Oita, 870-0192 Japan; 2 Department of Cardiovascular Surgery, Oita University Hospital, Oita, Japan

**Keywords:** Endoscopic-assisted aortic surgery, Descending aortic replacement, Paraplegia, Minimally invasive surgery

## Abstract

**Background:**

Descending aortic replacement often involves making large incisions; thus, it results in massive invasions. We report the case of a patient with dilated descending aorta treated using endoscopic-assisted descending aortic replacement with essentially minimal invasions.

**Case presentation:**

We performed endoscopic-assisted descending aortic replacement with a single incision involving six wounds by trocar puncturing on a 59-year-old man who was diagnosed with dilated descending aorta by stent graft-induced new entry. Subsequently, the patient was discharged on postoperative day 11 without any complications.

**Conclusions:**

Despite minor incisions, our approach can be indicated to almost the same group of patients in whom the conventional approach can be performed. Our procedure involved a single incision of only 8 cm and six wounds by trocar puncturing. Thus, endoscopic-assisted surgery can be a useful option in descending aortic surgery.

## Background

The application of endoscopic surgery is rapidly being established and expanded in cardiac surgery. However, the use of endoscopes in aortic surgeries remains limited. In this report, we present a case that involved the performance of endoscopic-assisted descending aortic replacement with a single incision of only 8 cm and six wounds by trocar puncturing. We consider it an important option for aortic surgeries to properly use endoscopes in this minimally invasive era.

## Case presentation

**Fig. 1:**
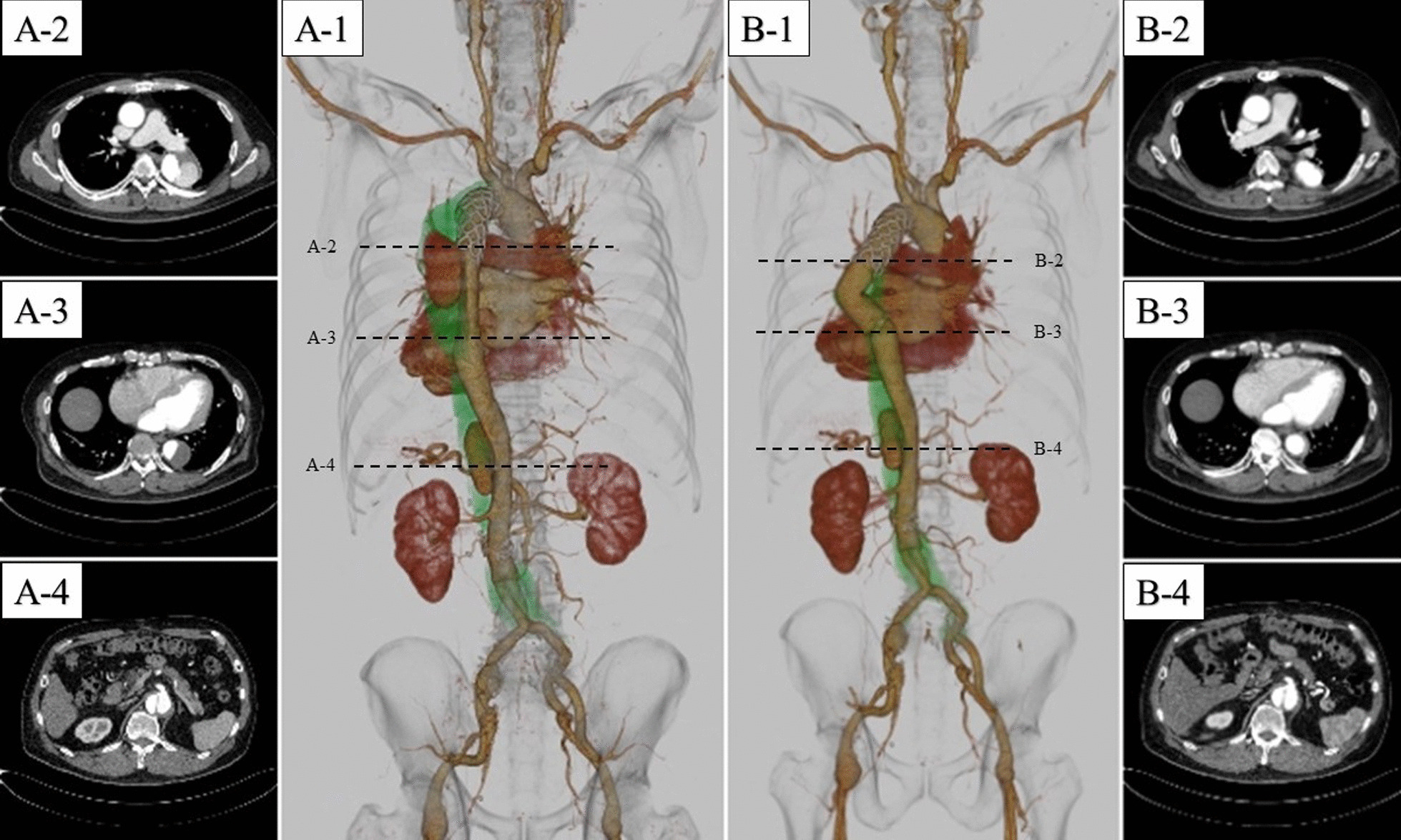
Preoperative (**A1–4**) and postoperative (**B1–4**) computed tomography. **A2** shows the stent graft-induced new entry lesion. **B2** shows that the entry lesion was successfully excluded

**Fig. 2:**
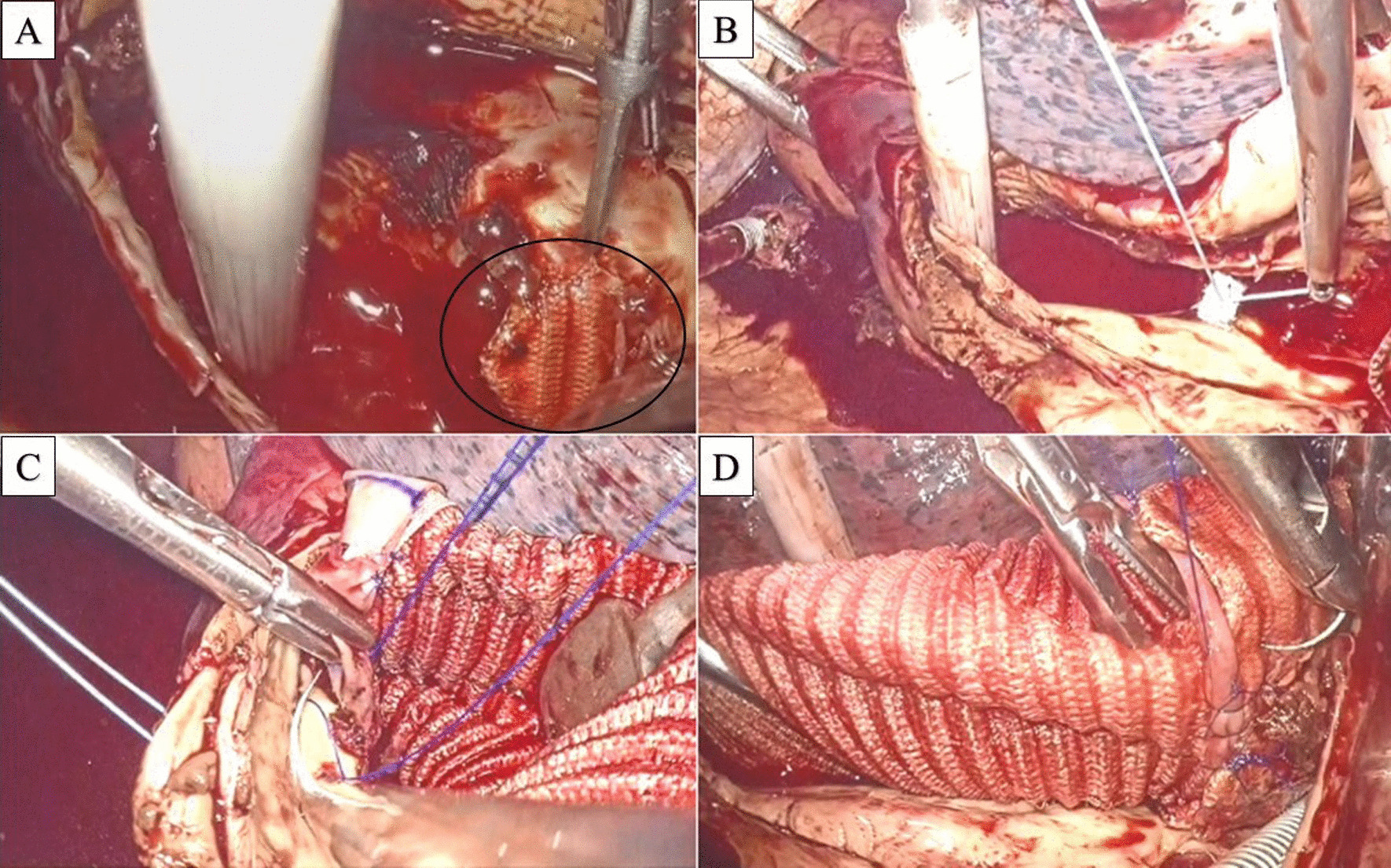
**A**–**D** show intraoperative images. **A** Circle shows the stent graft-induced new entry lesion. **B** Sealing intercostal arterial orifice. **C** Distal anastomosis by inclusion technique. **D** Proximal anastomosis

**Fig. 3:**
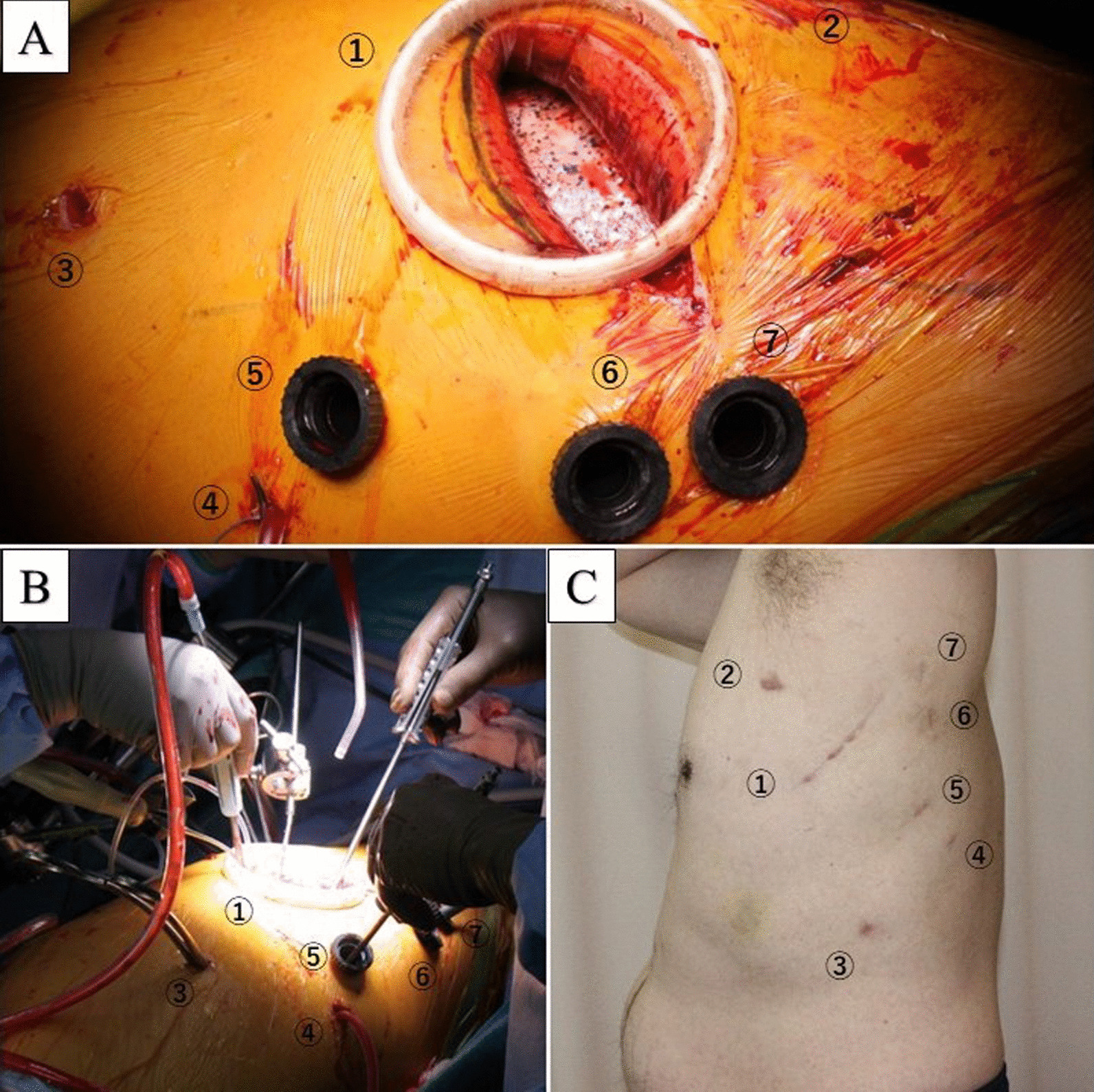
**A–C** show intraoperative and postoperative wound images, respectively. The numbers in **A–C** correspond to each. Main incision (1), proximal aortic clamp site (2), distal aortic clamp site (3), suction tube (4), trocar for endoscope, and devices (4–6)

A 59-year-old man was admitted to our hospital for progressive dilatation of the descending aorta. Anamnesis revealed no drug use or family history for the condition. One year before admission, he was diagnosed with type B aortic dissection while during the same hospitalization period, he was later diagnosed with acute myocardial infarction. The primary tear was located in the distal aortic arch. Open stent-grafting to seal the primary entry and coronary artery bypass grafting were performed. Three months later, his abdominal aorta was replaced with a Y-shaped graft due to dilation of his abdominal aorta. Thereafter, he received computed tomography (CT) follow-up regularly. The diameter of the descending aorta decreased for the first six months; however, CT defined redilatation of the descending thoracic aorta one year after surgery. Contrast-enhanced CT revealed the presence of stent graft-induced new entry (SINE). The maximum minor-axis diameter of the descending thoracic aorta was 52 mm (Fig. 1). Transthoracic echocardiography revealed a reduced ejection fraction, 37.1%, and inferior-lateral hypokinesis. This patient had already undergone open stent-grafting and Y-shaped grafting. We deduced that this patient was at high risk of paraplegia after conservative descending aortic replacement. Thoracic endovascular aortic repair (TEVAR) was considered; however, there were further SINE risks due to the patient’s history of type B aortic dissection and other aortic diseases. Thus, we decided to perform the procedure via minimal thoracotomy with a 3D endoscope to allow as much collateral circulation as possible. Cerebrospinal fluid drainage was performed on the day before the operation. The patient was placed in a modified right lateral decubitus position and the 5th intercostal space was opened by 8 cm and one-lung ventilation was started. Trocars for the IMAGE 1 SPIES 3D endoscope system (Karl Storz, Tuttlingen, Germany) and instruments were inserted through the 5th, 6th, and 7th intercostal spaces. Proximal and distal aortic clamps were inserted through the 4th and 9th intercostal spaces, respectively. Furthermore, a suction line was inserted through the 8th intercostal space (Figs. 2, 3). The patient underwent mild (35 °C) hypothermic partial cardiopulmonary bypass with the left femoral artery perfusion along with drainage of the right jugular and femoral veins. The endoscope was inserted through the 7th intercostal space for the proximal side procedure, and the 5th intercostal space for the distal side procedure. Surgical instruments were inserted through the main incision and another port separate from the endoscopic port. While carefully separating the descending aorta and avoiding injuring the esophagus, the proximal and distal aorta were clamped. Opening the descending aortic aneurysm revealed the entry tear due to an open stent graft (Fig. 2A). After sealing three intercostal arterial orifices (Fig. 2B), we performed distal anastomosis by a continuous-suture graft-inclusion technique (Fig. 2C) with a 26 mm J Graft SHIELD NEO® (Japan Lifeline Co., Ltd., Tokyo, Japan) using 3–0 polypropylene sutures with a bovine pericardium strip. Traction sutures for the aortic wall were applied to avoid sewing the esophagus. Subsequently, we performed proximal anastomosis directly to the open stent graft (Fig. 2D) using 4–0 polypropylene sutures. A bovine pericardium strip was sewn to the inside of the open stent graft without aortic wall. The cardiopulmonary bypass time was 179 min. He was discharged on the postoperative day 11 without any complications.

## Discussion

Even though minimally invasive cardiac surgery (MICS) spreads widely in the field of cardiac surgery, descending aortic replacement surgery still involves high invasiveness. The conventional approach requires making a large incision with transection of rib bones to gain a large surgical field. Since Dake et al. reported the first case of TEVAR for descending aortic aneurysm in 1994 [1], descending aortic treatment has changed in terms of low invasiveness in which it is no longer necessary to use one-lung ventilation or cardiopulmonary bypass. However, TEVAR is only advised to highly selective patients to whom it is anatomically feasible. Despite minor incisions, our approach can be indicated to almost the same group of patients in whom the conventional approach can be performed. In our case a single incision of only 8 cm and six wounds by trocar puncturing were performed.

There are several advantages associated with the use of an endoscope for aortic surgery. For instance, endoscopic surgery provides lower invasiveness and a clearer endoscopic view in a compatible way. Further, a 3D endoscope provides better depth perception and spatial orientation. In addition, smaller incisions result in lesser surgery-related blood loss and pain [2]. Consequently, patients are expected to stay for a shorter duration in the hospital. One limitation of endoscopic-assisted descending aortic surgery is the difficulty of reconstructing intercostal arteries. Intercostal arteries reconstruction and operative time are trade-offs; however, this problem is expected to solve as the surgeon performs a greater number of surgeries.

To minimize the risk of paraplegia after descending aortic surgery, various precautions have been taken [3–6]. As we have already reported that endoscopic-assisted aortic surgery might have reduced risk of spinal cord injury [7]. This is because preserving the intercostal arteries and multiple vessels surrounding the chest muscles is important to maintain the blood supply to the spinal cord [8]. Furthermore, large incisions can damage the intercostal arteries and multiple vessels running in the chest muscles. Our method may contribute towards maintaining the collateral blood network of the spinal cord and prevent paraplegia, especially in high-risk patients. Previously, we reported a similar case of endoscopic-assisted descending aortic replacement. However, that case was performed with an approximate incision of 30 cm while the incision in current case was only 8 cm. We believe that this reduction in the incision site from 30 to 8 cm will lead to totally endoscopic descending aortic surgery in the future.

## Conclusion

We report a case of endoscopic-assisted minimally invasive descending aortic surgery. Based on the outcomes noted in our patient, endoscopic-assisted surgery can be a useful option in descending aortic surgery. Our method can be used toward the development of totally endoscopic descending aortic surgery in the future.

## Data Availability

The datasets of this article are available on reasonable request.
